# Supplementary material for: Three-dimensional super-resolution fluorescence imaging of DNA
Source: Sci Rep. 2020 Jul 27;10:12504. doi: 10.1038/s41598-020-68892-5 (PMC7385144; doi:10.1038/s41598-020-68892-5)
Supplement: Supplementary file 1 — Supplementary Information [file 41598_2020_68892_MOESM1_ESM.pdf]

Supplementary information

Three-dimensional super-resolution fluorescence imaging of DNA

Sevim Yardimci, Daniel R. Burnham, Samantha Y. A. Terry, Hasan Yardimci

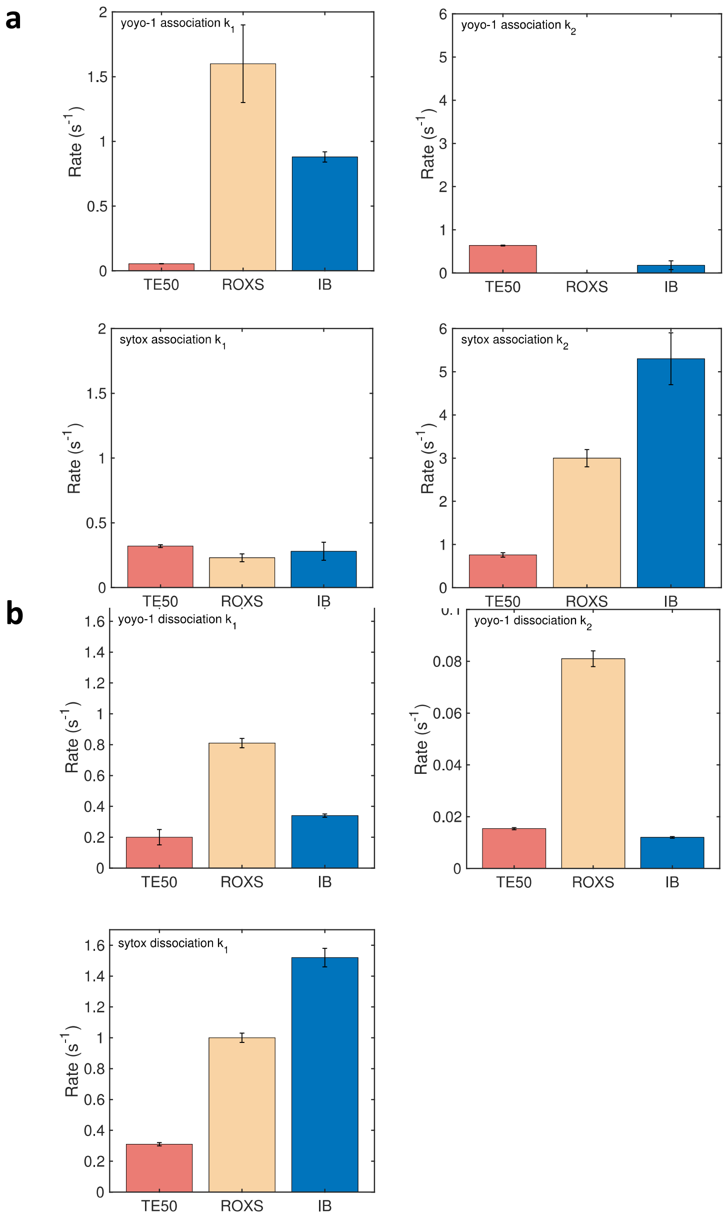

Supplementary Figure 1. Comparison of YOYO-1 and SxO association and dissociation kinetics. a, Kinetic parameters of association. Despite YOYO-1 having a higher rate of reaction for free dye to DNA bound dye, k_1, the much faster autocatalysis of DNA bound dye, k_2, results in faster association for SxO. **b,** Kinetic parameters of dissociation. Dissociation of SxO is faster mostly due to the lack of a second, slow, kinetic step.

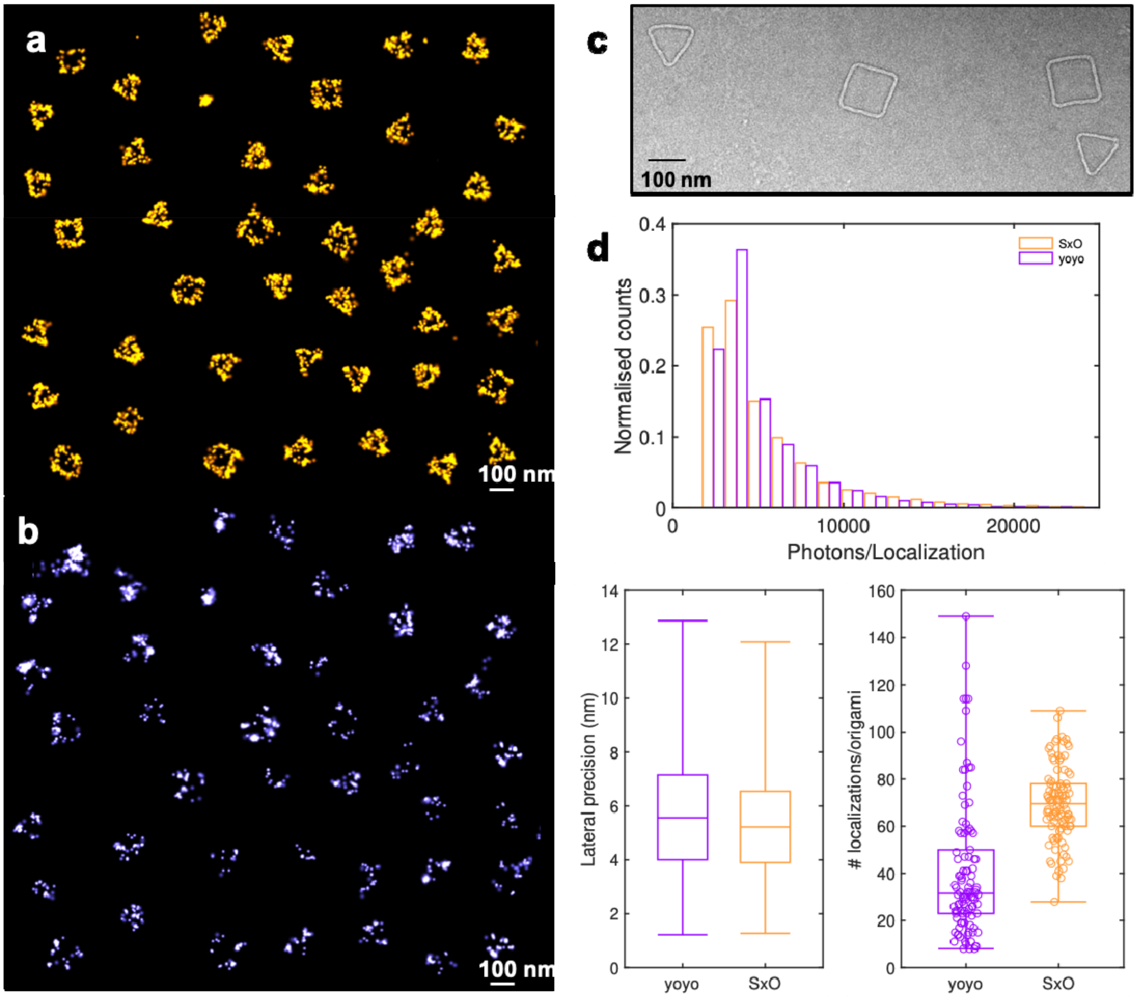

Supplementary Figure 2. 2D BALM images of DNA origami with edge length of 120 nm. a-b, Super-resolution imaging of triangular and square-shaped DNA origami with DNA-intercalating dye 50 pM SxO (**a**) or 200 pM YOYO-1 (**b**). All images were reconstructed from localisations in 10500 frames at 50ms exposure. The images were constructed using molecules from multiple fields of view. **c,** Transmission electron microscopy images of DNA origami molecules. **d,** Statistics of localizations comparing yoyo and SxO. Histogram of number of photons per localization. The number of photons per localisations are 3387 ± 47 and 5104 ± 44 (mean ± sem) for yoyo and SxO, respectively. Box plot for lateral precision of both YOYO-1 and SxO data, and bee-swarm overlaid box plot for number of localizations per origami. Box plots show median, 25th and 75th percentiles and minimum and maximum values.

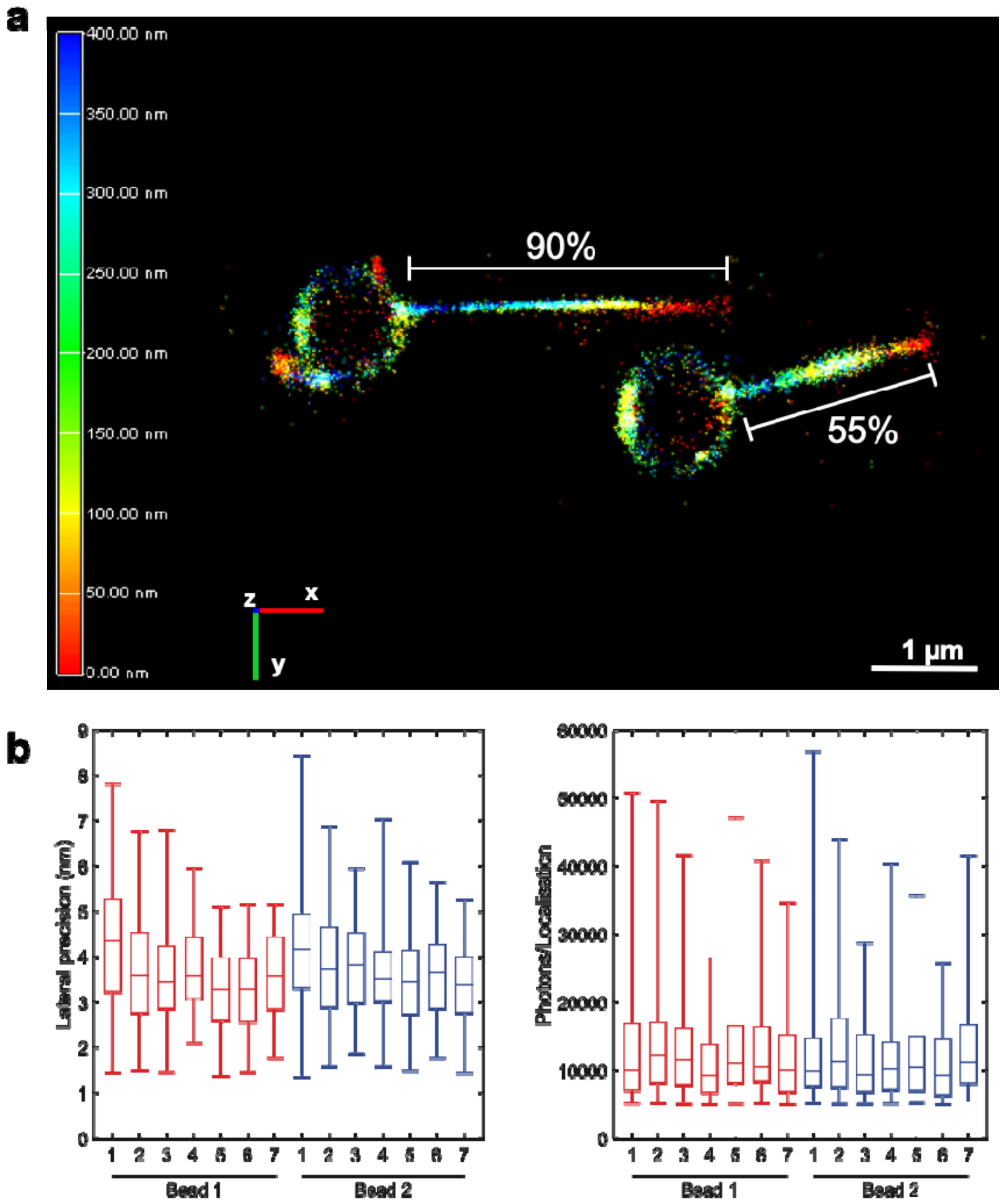

Supplementary Figure 3. 3D BALM images of linear DNA molecules and statistics of localisations for Fig. 2c. a, BALM images of 10 kb DNA constructs stretched between the surface of the glass slide and 1 μm bead. The width of a DNA molecule stretched to 55% of its contour length is larger than that of 90% stretched DNA due to thermal fluctuations of the DNA molecule. **b,** Lateral precision and photons per localization for data in Fig. 2c. Here we plot the statistics for each of the seven axial layers imaged for both microspheres. Box plots show median, 25th and 75th percentiles and minimum and maximum values. The number of photons per localisations are 12878 ± 277 and 12240 ± 259 (mean ± sem) for beads 1 and 2, respectively.

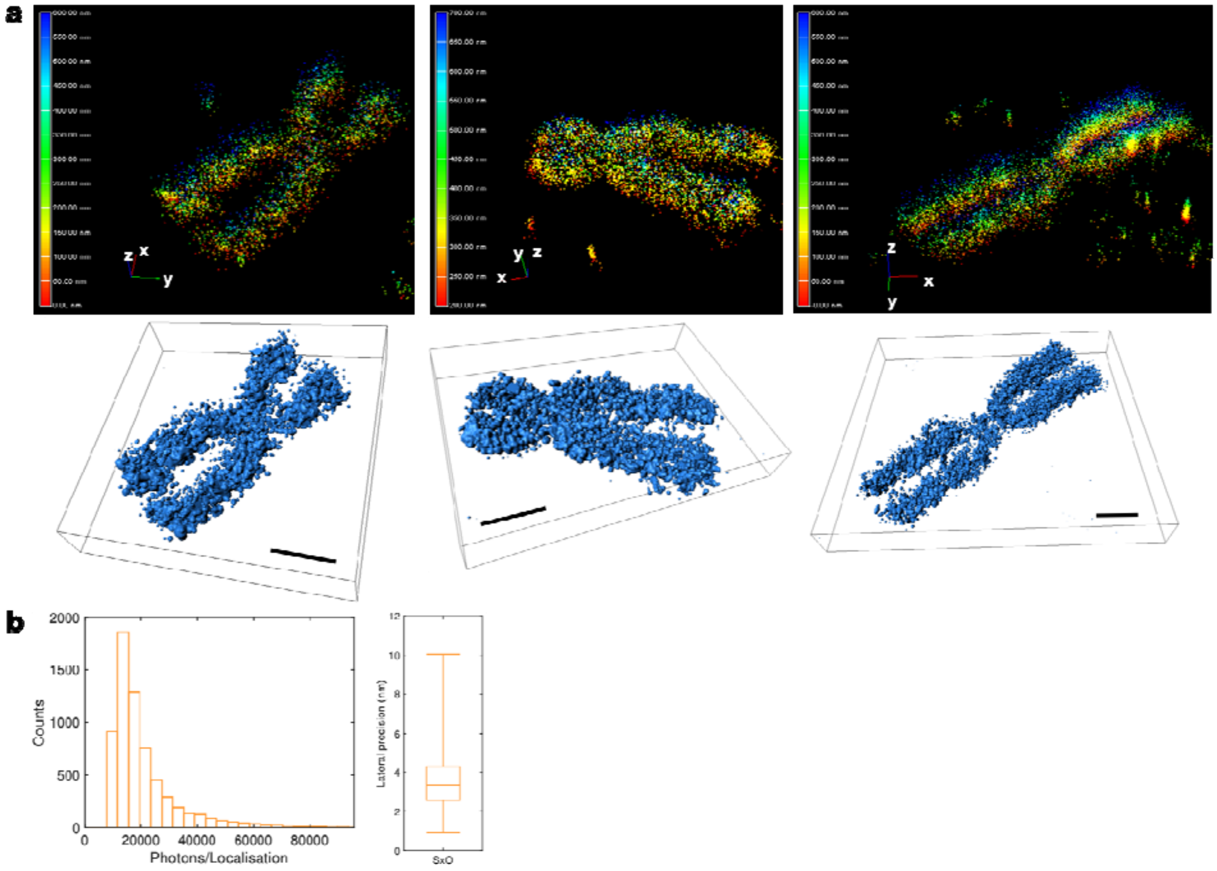

Supplementary Figure 4. 3D BALM images of metaphase chromosomes and associated localization statistics. a, Three-dimensional BALM images of human chromosomes and (bottom) representing three-dimensional surface plots. Scale bars: 1 μm. **b,** Histogram of the number of photons per localization and box plot of the localization lateral precisions for the left most chromosome in **a**. Box plots show median, 25th and 75th percentiles and minimum and maximum values. The data for this figure has been truncated for lateral precisions greater than 10nm. The number of photons per localisations are 20592 ± 151 (mean ± sem).

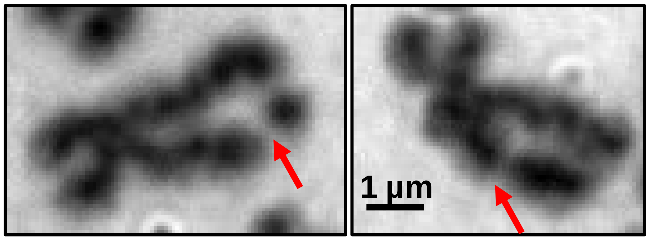

Supplementary Figure 5. Sample metaphase chromosomes with chromatid breaks. Bright-field images of 1Gy irradiated metaphase chromosomes. Red arrows show the chromatid gaps.

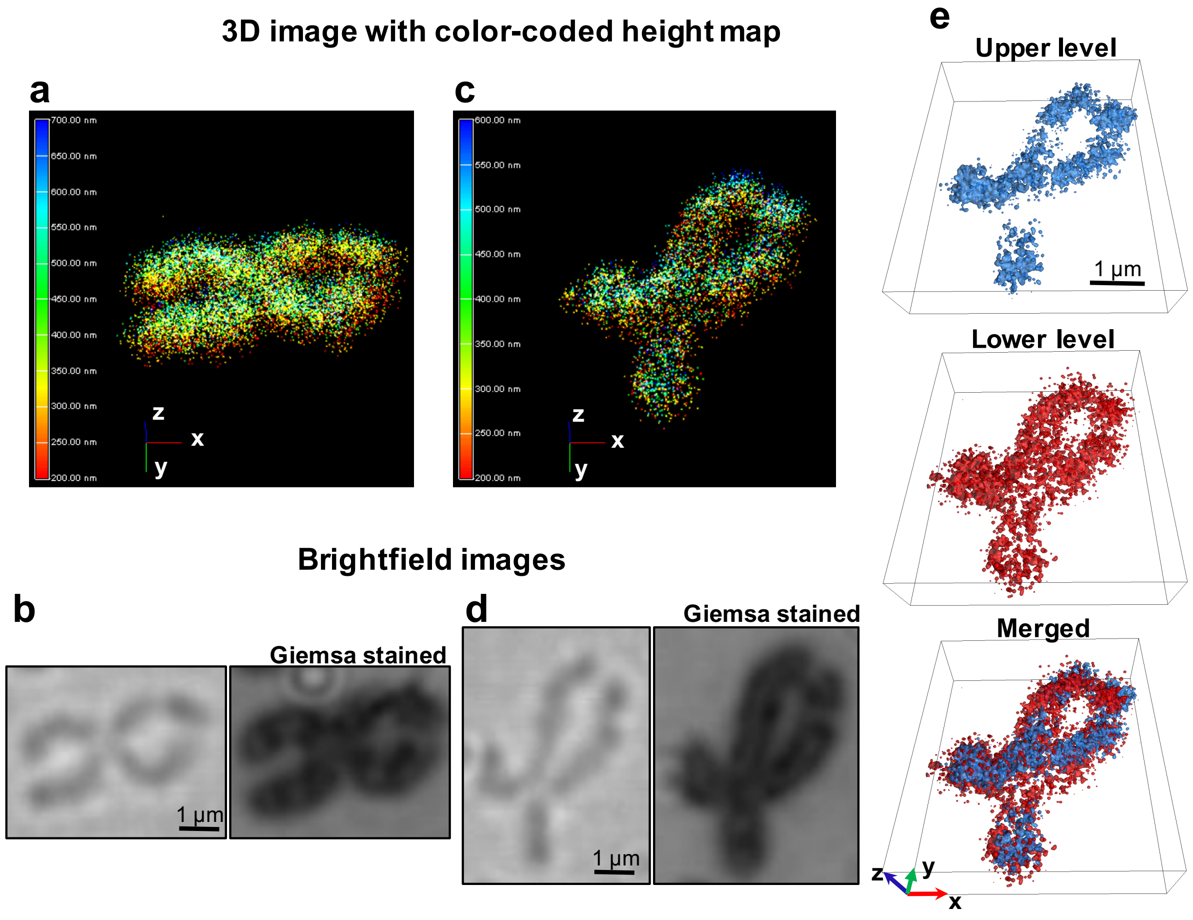

Supplementary Figure 6. 3D super-resolution imaging of irradiated metaphase chromosomes. **a**, Three-dimensional BALM image of human chromosome (same chromosome shown in Fig. 2b) with a break as a result of radiation with color-coded height map and **b**, the corresponding bright-field image of the same chromosome before (top) and after (bottom) Giemsa staining. **c,** Another example of 3D BALM image of irradiated human chromosome containing a gap and **d,** its bright-field image of before (left) and after (right) Giemsa staining. **e,** Representing three-dimensional surface plot of the chromosome in **c** at upper (blue), lower (red) z planes and bottom merged images.

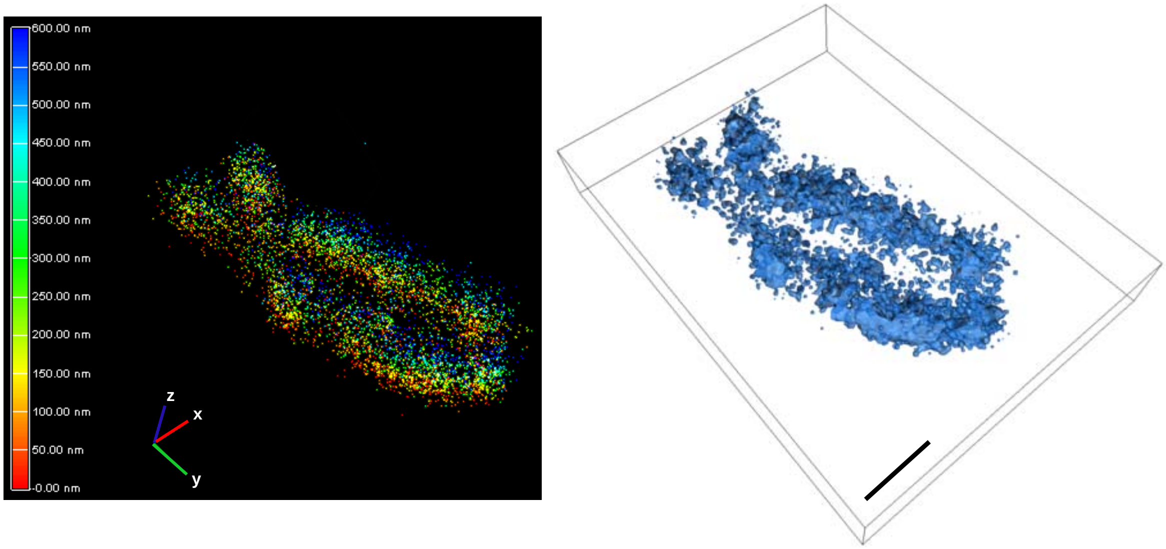

Supplementary Figure 7: 3D BALM imaging of sonication-induced DNA damage. 3D BALM image of a sonication-treated metaphase chromosome (left) and the corresponding three-dimensional surface plot (right). Scale bar: 1 µm.

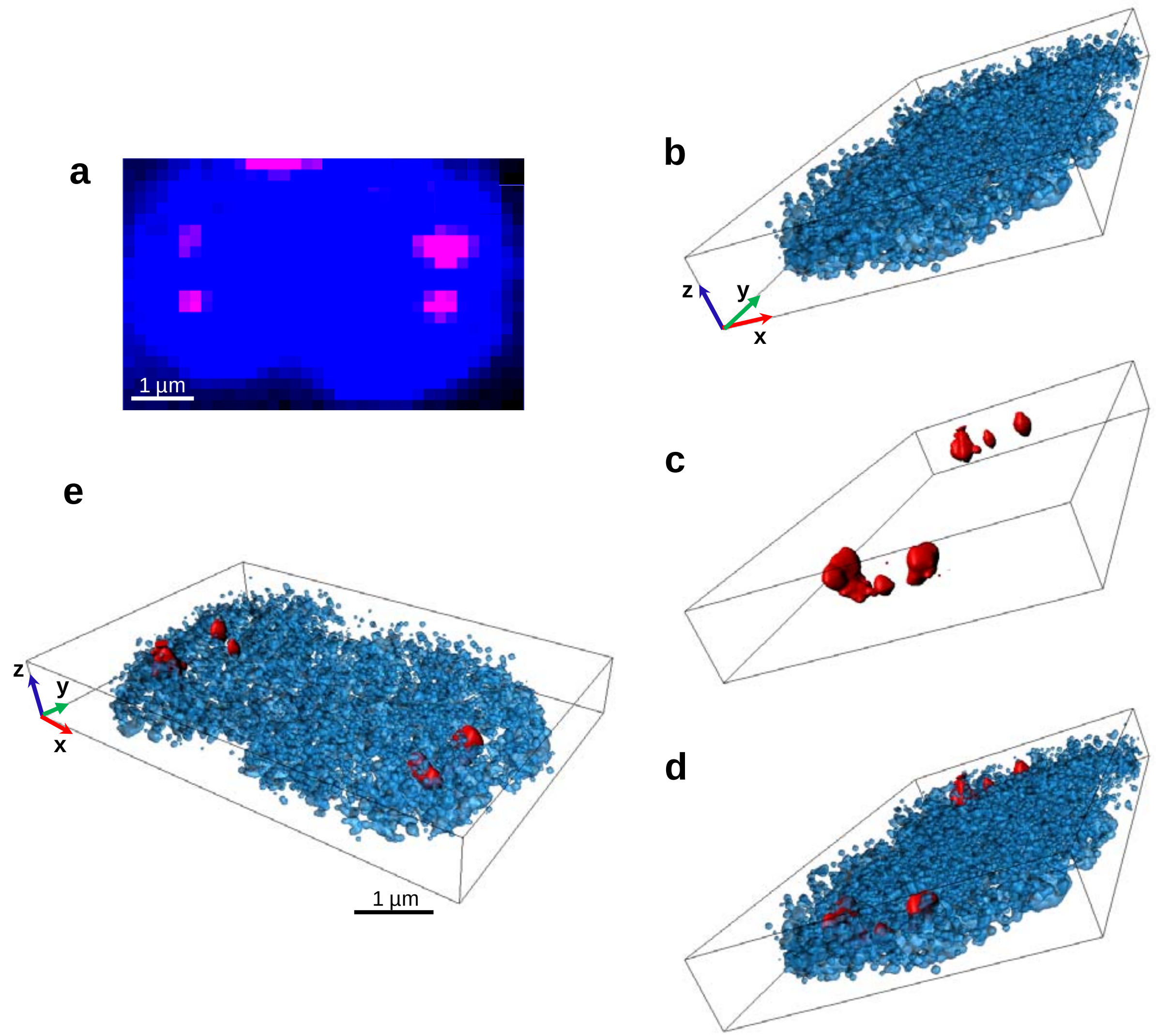

Supplementary Figure 8. 3D BALM on metaphase human chromosomes and 3D STORM on telomeric regions. a, Merged wide-field image of a human metaphase chromosome (blue) and telomeric regions (magenta). **b,** 3D BALM image of the same chromosome. **c,** 3D STORM image of telomeric regions on the chromosome. **d-e,** Composite 3D BALM and STORM images of the chromosome and the telomeric regions on the chromosome.

	I_0 (a.u.)	k_1 (s^{-1})	k_2 (s^{-1})	A_1 (a.u.)	A_2 (a.u.)
YOYO Dissociation					
TE50	-	0.20 ± 0.05	0.0154 ± 0.0004	0.071 ± 0.010	0.92 ± 0.01
ROXS	0.294 ± 0.002	0.81 ± 0.03	0.081 ± 0.003	0.387 ± 0.007	0.341 ± 0.005
IB	-	0.34 ± 0.01	0.0120 ± 0.0003	0.439 ± 0.006	0.561 ± 0.004
YOYO Binding					
TE50		0.055 ± 0.001	0.636 ± 0.008		
ROXS		1.6 ± 0.3	6E-7 ± 0.7		
IB		0.88 ± 0.04	0.18 ± 0.10		
SYTOX Dissociation					
TE50	0.15 ± 0.010	0.31 ± 0.01		0.80 ± 0.01	
ROXS	0.028 ± 0.004	1.00 ± 0.03		1.07 ± 0.02	
IB	-0.011 ± 0.005	1.52 ± 0.06		1.16 ± 0.03	
SYTOX Binding					
TE50		0.32 ± 0.01	0.76 ± 0.05		
ROXS		0.23 ± 0.03	3.0 ± 0.2		
IB		0.28 ± 0.07	5.3 ± 0.6		

Supplementary Table 1. Values of kinetic parameters from the model fits in Figure 1. a.u. are arbitrary units.
